# Effects of Powdered and Granular AMF on Maize Growth Under Low Fertilizer Conditions

**DOI:** 10.3390/jof12020123

**Published:** 2026-02-09

**Authors:** Ye Yuan, Zhengjun Feng, Huiping Song, Ao Yuan, Le Chang, Yan Zou, Munkhbat Dashdorj, Zhiwei Bian

**Affiliations:** 1 Institute of Resources and Environmental Engineering, Shanxi University, Taiyuan 030006, China; 2 Shanxi Qinghuan Nengchuang Environmental Protection Technology Company Limited, Taiyuan 030006, China; 3 School of Engineering and Technology, Mongolian University of Life Sciences, Ulaanbaatar 17029, Mongolia; dashdorj@mncea.edu.mn; 4 China-Mongolia Belt and Road Joint Laboratory of Mineral Processing Technology, Inner Mongolia Academy of Science and Technology, Hohhot 010000, China

**Keywords:** reduced fertilization, arbuscular mycorrhizal fungi, microbial functional groups, economic benefits, sustainable agriculture

## Abstract

Excessive fertilizer use drives soil degradation and resource waste. This study investigates how arbuscular mycorrhizal fungi (AMF) formulations (powder vs. granular) optimize maize (*Zea mays* L.) yield, soil microbiome, and economic benefits under 50% and 75% fertilizer reduction. Field trials showed that the AMF powder formulation under 50% fertilizer reduction (AP50) increased maize yield by 14.67%. This increase was associated with rapid root colonization (85.3%), enhanced phosphorus availability, and the recruitment of beneficial fungi such as *Mortierellomycota*. Granular formulation at 75% reduction (AG75) achieved 7.18% yield gain via sustained symbiosis. Fungal communities exhibited greater sensitivity to fertilization than bacteria (Chao1, *p* = 0.0094), with AMF suppressing *Fusarium* by 42% while enriching functional taxa (*Actinobacteria*, *Mortierellomycota*). Economic analysis confirms that AP50 (30,435 CNY/ha) and AG75 (26,954 CNY/ha) yield higher net profits, where CNY denotes Chinese Yuan. Powder formulations maximize immediate benefits in medium- to low-fertility soils, whereas granules support long-term soil health in high-organic systems, providing a precision strategy for sustainable agriculture.

## 1. Introduction

Global agriculture faces the dual challenge of ensuring food security while mitigating soil degradation and environmental pollution caused by excessive fertilizer use [1]. In China, fertilizer application rates are 1.5 times higher than the global average, leading to widespread soil acidification and increased production costs [2,3]. For maize cultivation, fertilizer constitutes 20–30% of total input costs, yet indiscriminate reduction can cause significant yield losses [4]. Therefore, developing strategies that maintain productivity while reducing fertilizer dependency is essential for sustainable agriculture.

Current research often focuses on short-term agronomic effects, overlooking long-term microbial community dynamics under reduced fertilization [5,6,7]. Fertilization regimes profoundly influence soil microbial structure and function: excessive chemical fertilizers reduce microbial diversity and may promote pathogenic fungi, whereas balanced reduction can enhance fungal abundance and functional stability [8,9,10].

Arbuscular mycorrhizal fungi (AMF), due to their key roles in nutrient uptake, stress resistance, and microbial interactions, are considered a pivotal biotechnological alternative to chemical fertilizers [11]. AMF significantly enhance plant nutrient use efficiency, improve soil structure, and promote microbial diversity [12]. Numerous studies have shown that AMF inoculation under reduced fertilization improves soil structure [13], increases crop yield [14], and modulates microbial community structure [10]. Recent analyses of commercial AMF inoculants reveal that most products employ solid formulations—predominantly powder (around 65%) and granular (around 25%)—with propagule viability and carrier properties directly affecting colonization efficiency and long-term field performance, particularly under varying fertilization regimes [15,16]. These formulation differences can influence initial propagule delivery, survival, and subsequent interactions with native soil microbiota, though direct comparative field trials remain scarce [17].

Moreover, economic feasibility analyses are scarce. Existing studies have not addressed whether the increased cost of powder inoculants (35–50% higher, ~150–220 CNY/ha) can be offset by yield gains, or whether the reduced yield during symbiosis establishment with granules offsets their cost reductions. Additionally, long-term benefits such as soil health improvement (organic matter increase of 0.2–0.5% per year) and fertilizer reduction potential (N/P_2_O_5_ cut by 20–30%) remain to be quantified to support farmer adoption. Hence, an integrated evaluation framework incorporating formulation optimization, microbial functional responses, and economic feasibility is urgently needed to resolve the decision-making dilemma for large-scale AMF application. This cost–benefit trade-off directly influences farmer adoption and scalability.

Thus, this study aims to evaluate the effects of different AMF formulations on maize yield, soil microbial communities, and economic benefits under reduced fertilization. Notably, this is among the first field-based studies to directly compare the agronomic and ecological performances of powder versus granular AMF inoculants under fertilizer reduction scenarios, while integrating high-throughput microbial community analysis with a detailed economic assessment. By incorporating microbial functional dynamics and cost–benefit evaluation, we seek to identify sustainable cropping models that support the transition toward green agriculture, and to provide actionable insights for farmers and policymakers.

## 2. Materials and Methods

### 2.1. Experimental Site and Design

The field experiment was conducted in Shangfu Village (SF), Yonglu Township, Gaoping City, Shanxi Province, China (112.89° E, 35.89° N). The site is characterized by a warm temperate semi-humid continental climate, with an elevation of 1186.8 m, an average annual temperature of 10 °C, and maximum and minimum temperatures of 35 °C and −15 °C, respectively. Mean annual precipitation is approximately 550 mm, concentrated mainly from July to September. The frost-free period lasts about 180 days, which is favorable for maize growth and maturation. The field experiment was conducted during the 2024 maize-growing season in Shangfu Village. Field preparation and basal fertilizer application were completed in late April 2024. Maize sowing, along with the application of AMF inoculants and the remaining fertilizer, was carried out on 28 April 2024. The maize growth period spanned April to September 2024, with final harvest and yield measurement conducted on 20 September 2024. The soil properties of the experimental site are shown in Table 1.

The AMF inoculant (powder and granular formulations, Shanxi Gerun Hetai Co., Ltd., Taiyuan, China) contained a consortium of five species adapted to local conditions: *Claroideoglomus etunicatum*, *C. claroideum*, *Rhizophagus irregularis*, *Funneliformis geosporus*, and *F. mosseae*. The powder formulation had a mean propagule concentration of 6.10 × 10^5^ infective units per kg, with a carrier based on milled expanded clay and zeolite. The granular formulation had a mean concentration of 3.25 × 10^5^ units per kg, using expanded clay and zeolite granules as the carrier. The maize cultivar “Qiangsheng 192”, widely cultivated and well-adapted to the local semi-arid climate and soil conditions of the Shanxi region, was selected for this study. This hybrid is known for its high yield stability and moderate nutrient requirement, making it a representative model for assessing agronomic interventions under reduced fertilization.

In this study, there were nine fertilizer application methods, each of which was repeated three times with distinct boundaries established between plots. Compound fertilizer (N-P_2_O_5_-K_2_O: 15-15-15) was used as the base fertilizer. The total application rate for the full-fertilizer treatment (100%) was 600 kg/ha, which supplied 90 kg of N/ha, 90 kg of P_2_O_5_/ha, and 90 kg of K_2_O/ha. Fertilizer was applied at a rate of 600 kg/ha, 75% fertilizer was applied at a rate of 450 kg/ha, 50% fertilizer was applied at a rate of 300 kg/ha, AMF powder was applied at a rate of 0.225 kg/ha and was sown by mixing it to adhere to the surface of the seeds, and AMF granules were applied at a rate of 15 kg/ha and were hole-applied at sowing time near the maize seed. The AMF granules were applied at a rate of 15 kg/ha and were hole-applied near the maize seeds during sowing. This study employed a 3 × 3 factorial design, establishing nine fertilization treatments comprising three chemical fertilizer application rates (50%, 75%, and 100% of the conventional recommended rate) and three arbuscular mycorrhizal fungi (AMF) inoculation methods (inoculation with AMF powder, inoculation with AMF granules, and an uninoculated control). Treatment combinations were clearly coded as follows: AP50, AP75, and AP100 (corresponding to AMF powder inoculation at three chemical fertilizer application rates); AG50, AG75, and AG100 (corresponding to AMF granule inoculation at three chemical fertilizer application rates); and CK50, CK75, and CK100 (corresponding to the control without AMF inoculation at three chemical fertilizer application rates).

Based on the five-point sampling method [18], we collected 200 g of soil samples and mixed them thoroughly using the quartering method. For each treatment plot, the five collected sub-samples were uniformly mixed in the field to form a composite sample representative of that plot. The collected soil constituted rhizosphere soil. At maize harvest, plants were carefully dug up, large clods of loose soil were shaken off, and soil tightly adhering to the root surface (approximately 0–4 mm in depth) was collected using a sterile brush.

### 2.2. Analytical Methods

#### 2.2.1. Soil Physicochemical Properties Analysis

Soil pH was measured in a soil-water suspension at a 1:2.5 ratio using a pH meter. Electrical conductivity (EC) was determined following the saturated paste method [17]. Soil organic matter (SOM) content was assessed by the potassium dichromate oxidation method as an indicator of soil fertility [19]. Total nitrogen (TN) was analyzed using the Kjeldahl digestion method. Total phosphorus (TP) was extracted using NaHCO_3_ and quantified by inductively coupled plasma optical emission spectrometry (ICP-OES 6300) (Thermo Fisher Scientific, Waltham, MA, USA). Total potassium (TK) was also measured by ICP-OES [20].

#### 2.2.2. Maize Yield Analysis

Maize was harvested from each plot, and the fresh weight was recorded. Moisture content was determined to convert fresh weight to dry weight. Yield was calculated using the following formula:

Y = 10,000/S × W × (1 − M)(1)

where Y is the yield (kg·ha^−1^), S denotes the area of the experimental field (m^2^), W denotes the total mass of maize at harvest (kg), and M denotes the moisture content of maize (%).

#### 2.2.3. Mycorrhizal Colonization Analysis

Roots were washed with deionized water, and fresh root subsamples were collected for mycorrhizal colonization assessment using the gridline intersect method [21]. At harvest, fine root segments (approximately 1 cm in length) were randomly sampled from three representative plants per plot. Root samples were cleared with 10% KOH and stained with trypan blue following the protocol of Phillips and Hayman. Mycorrhizal colonization was assessed using the gridline intersect method under a compound microscope at 200× magnification. For each plot, a minimum of 100 root intersects were examined, and the percentage of root length colonized by AMF structures (hyphae, arbuscules, or vesicles) was calculated.

#### 2.2.4. Microbial Community Analysis

DNA extraction was executed utilizing the EZNA Soil DNA Kit (Omega Bio-Tek, Norcross, GA, USA) in strict accordance with the manufacturer’s guidelines. The integrity of the DNA was evaluated through quantitative assessment and qualitative analysis via NanoDrop 2000 (Thermo Fisher Scientific, Wilmington, DE, USA) and 1% agarose gel electrophoresis, respectively, to scrutinize the concentration, quality, and purity of the isolated DNA. PCR amplification was conducted employing ds 338F and 806R primers specific to the bacterial V3–V4 region, and ITS1F and ITS2R primers tailored for the fungal ITS hypervariable region. The amplified products were sequenced on an Illumina NovaSeq 6000 platform (Shanghai Personalbio Technology Co., Ltd., Shanghai, China) to generate 250-bp paired-end reads. Raw sequencing data were processed using QIIME2. Briefly, reads were quality-filtered, denoised, merged, and chimera-removed to obtain amplicon sequence variants. Taxonomy was assigned against the SILVA database (release 138) for bacteria and the UNITE database (release 9.0) for fungi.

#### 2.2.5. Statistical Analysis

All statistical analyses were performed using R software (version 4.3.1) and DPS (Data Processing System) software (version 17.0). Data are presented as mean ± standard deviation (SD) unless otherwise stated, with the number of biological replicates (*n*) specified in the figure legends or results text. For soil physicochemical properties and maize yield data, one-way analysis of variance (ANOVA) was first applied to assess the overall effect of treatments. When the ANOVA indicated a significant effect, Duncan’s multiple range test was employed for post-hoc comparisons among treatment means. Significant differences between treatments are denoted by different lowercase letters in the figures and tables. To assess the diversity within microbial communities (α-diversity), indices including Chao1 (richness), Shannon (diversity), Simpson (dominance), and Good’s coverage were calculated. Differences in these indices among treatments for bacterial and fungal communities were compared using Student’s *t*-test (for comparisons between two groups) or one-way ANOVA followed by Duncan’s test (for multi-group comparisons), as appropriate. The results of these comparisons are reported with exact *p*-values in the text.

To evaluate the compositional differences between microbial communities (β-diversity), principal coordinate analysis (PCoA) was performed based on Bray–Curtis’s dissimilarity matrices calculated from the amplicon sequence variant (ASV) tables. The statistical significance of observed clustering patterns among treatment groups was tested using permutational multivariate analysis of variance (PERMANOVA) with 999 permutations and implemented via the adonis2 function in the R vegan package (version 2.6-4). Results are reported with R^2^ and *p*-values.

The relationships between microbial community composition (based on ASV data) and soil environmental variables were examined using Mantel tests (Pearson correlation method with 999 permutations). The analysis was conducted separately for bacterial and fungal communities in the AMF powder (AP) and granular (AG) treatment series. Results are visualized with network diagrams where edge width corresponds to the Mantel r statistic and color indicates statistical significance. All data visualizations, including bar charts, scatter plots, heatmaps, and PCoA plots, were generated using ggplot2 (version 3.5.0) and pheatmap packages (version 1.0.12) in R (version 4.3.1) or OriginPro (version 2023). Significance levels are denoted as follows: *p* < 0.05, *p* < 0.01, *p* < 0.001; *p*-values between 0.05 and 0.10 are reported as trends.

## 3. Results

### 3.1. Effects of Fertilization Treatments on Soil Physicochemical Properties

**Figure 1:**
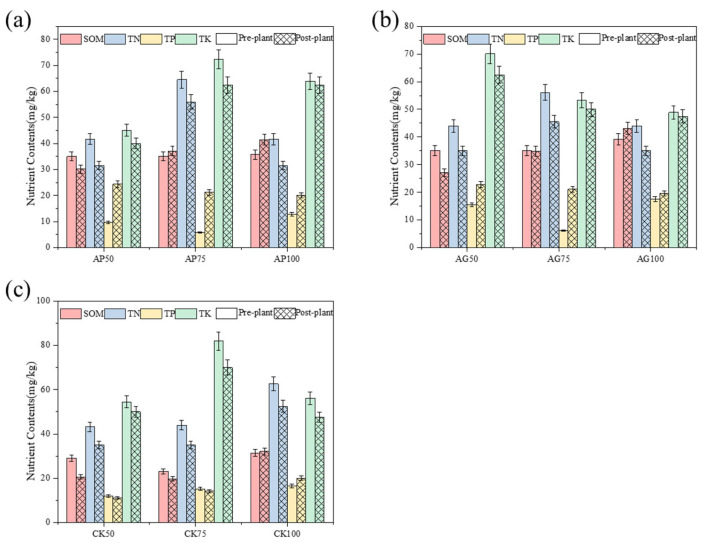
Changes in soil nutrient contents before and after maize cultivation under different fertilization treatments: (**a**) AMF powder inoculant treatments, (**b**) AMF granular inoculant treatments, (**c**) Control treatments without AMF.

Following maize cultivation, Figure 1 reveals that in soils treated with AMF powder and granular inoculants, alkaline hydrolysable nitrogen content decreased significantly, whilst available phosphorus content increased markedly. Changes in available potassium and organic matter were not statistically significant. In control groups (CK), total organic matter and available phosphorus increased only under full fertilization, whereas other nutrient components declined. Notably, the AMF powder treatment at 75% fertilization (AP75) exhibited the strongest improvement in total soil nutrients, indicating its potential for optimizing soil nutrient status. Correlation analysis between soil nutrient contents and fertilization levels in CK treatments revealed a positive relationship, but post-cultivation nutrient levels remained lower than pre-cultivation, suggesting further optimization of fertilization management is necessary to enhance soil fertility and crop productivity.

### 3.2. Mycorrhizal Colonization and Maize Yield

**Figure 2:**
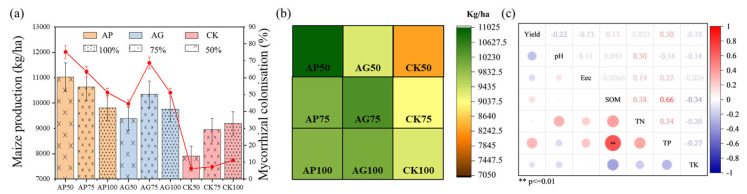
Effects of different fertilization treatments on maize yield: (**a**) Maize yield and mycorrhizal colonization rate, (**b**) Spatial distribution of maize yield, (**c**) Correlation analysis between yield and soil factors. Color gradient represents the Pearson correlation coefficient (r), from blue (negative correlation) to red (positive correlation).

This study evaluated the effects of AMF powder and granular inoculants on maize yield (Figure 2). Both AMF formulations significantly enhanced maize yield relative to their corresponding non-inoculated controls; colonization rates were significantly higher in all AMF-inoculated treatments (AP and AG series) compared to their corresponding non-inoculated control CK series (Figure 2a). The most substantial yield increases were achieved by the powder formulation under 50% fertilizer reduction (AP50, +14.67%) and the granular formulation under 75% reduction (AG75, +7.18%). The spatial distribution of yield across experimental plots further illustrated the consistent positive effect of AMF inoculation, particularly in the AP50 and AG75 treatments (Figure 2b). Correlation analysis revealed that yield was strongly and positively associated with key soil fertility indicators, most notably SOM and AP (Figure 2c).

### 3.3. α-Diversity of Soil Bacterial and Fungal Communities Under Fertilization Treatments

**Figure 3:**
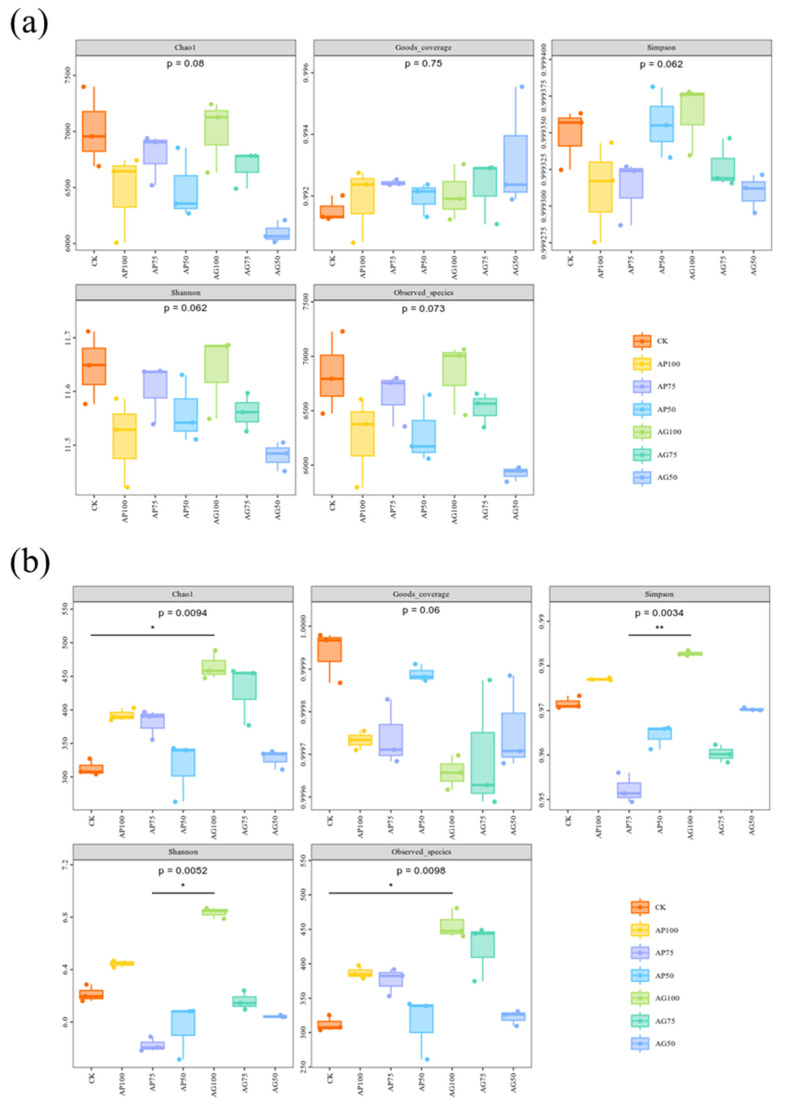
α-Diversity indices of soil microbial communities under different fertilization regimes: (**a**) Bacterial communities, (**b**) Fungal communities. Asterisks indicate significance levels: * *p* < 0.05, ** *p* < 0.01.

For bacterial communities (Figure 3a), most diversity indices did not reach statistical significance, although a trend suggested that fertilization might influence community composition and structure. The CK group exhibited relatively lower species richness and evenness. Fertilization and AMF inoculation significantly affected the α-diversity indices of fungal communities but had minimal impact on bacterial diversity indices. For fungal communities (Figure 3b), fungal richness (Chao1 index) was significantly higher in the AP75 and AG100 treatments compared to the CK group (*p* < 0.01). Good’s coverage index approached significance (*p* = 0.06) but showed less pronounced differences across treatments. Simpson index results (*p* = 0.0034) indicated significant effects of fertilization on fungal community heterogeneity, with AP75 and AG100 having higher evenness, reflecting a more balanced fungal distribution. The Shannon diversity index (*p* = 0.0052) also showed significant increases in fungal diversity under fertilization, particularly in AP75 and AG100 treatments compared with CK. Observed species counts were significantly higher in fertilized groups (*p* = 0.002). This metric represents the actual number of distinct ASVs detected per sample, complementing the Chao1 estimator of richness.

### 3.4. β-Diversity of Soil Bacterial and Fungal Communities Under Fertilization Treatments

**Figure 4:**
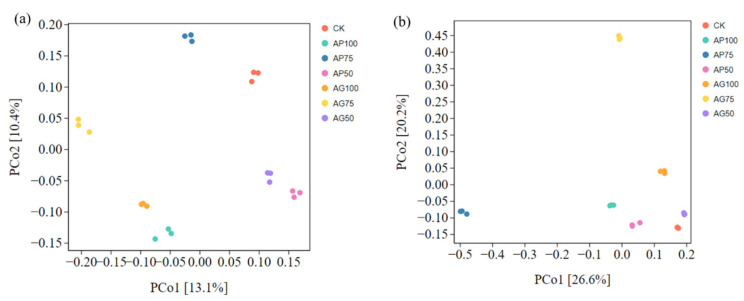
Principal Coordinate Analysis (PCoA) of soil microbial community β-diversity: (**a**) Bacterial communities, (**b**) Fungal communities.

Principal Coordinate Analysis (PCoA) was conducted to evaluate the differences in microbial community structure across fertilization treatments (Figure 4). For bacterial communities (Figure 4a), distinct clustering patterns were observed according to fertilization regime. CK samples clustered tightly in the lower left quadrant, with higher fertilization levels in the AP100 and AP75 treatments forming a distinct separation from the CK group. Granular treatments AG100 and AG75 also exhibited elevated bacterial diversity but showed distinct clustering from powder treatments, suggesting fertilization type influences bacterial community composition. Lower fertilization levels in AP50 and AG50 resulted in less pronounced community differences. For fungal communities (Figure 4b), similar clustering trends were identified. CK samples displayed limited diversity with tight clustering, whereas AP100 and AP75 treatments were clearly distinguishable from the control group CK.

### 3.5. Relative Abundance of Soil Bacterial and Fungal Communities Under Different Fertilization Treatments

**Figure 5:**
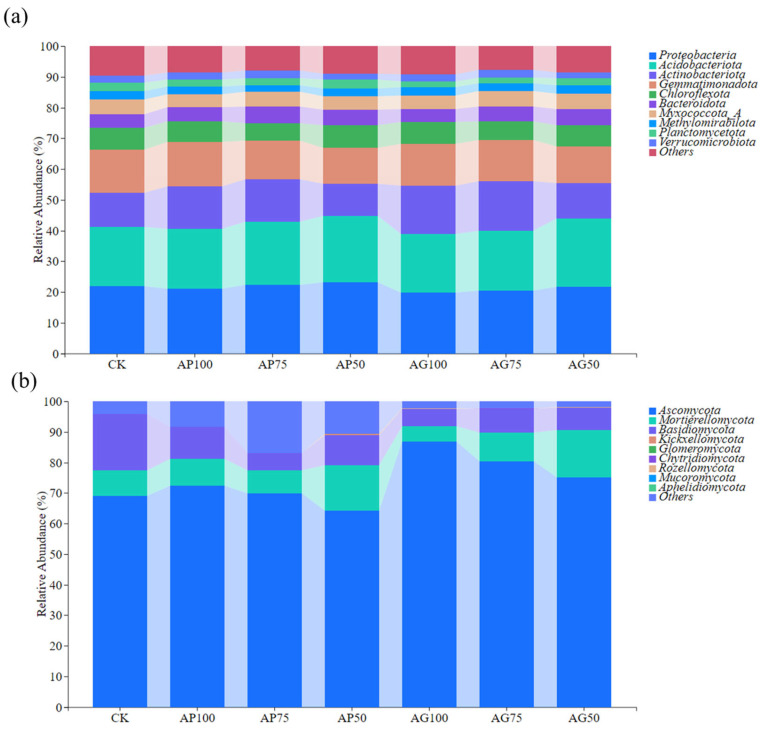
Relative abundance of soil microbial communities at the phylum level under different fertilization treatments: (**a**) Bacteria, (**b**) Fungi.

The relative abundance of microbial communities at the phylum level was analyzed across treatments (Figure 5). In bacterial communities (Figure 5a), *Proteobacteria* was the dominant phylum across all treatment groups. Its relative abundance was highest in the non-inoculated control groups (CK series). With increasing fertilizer application in the AMF powder treatments, particularly in AP100 and AP75, the relative abundance of *Proteobacteria* decreased, while the abundances of *Actinobacteria* and *Chloroflexi* increased. Similar trends were observed in the AMF granular treatments (AG100 and AG75). In contrast, bacterial community composition at the phylum level showed smaller shifts under the lower fertilization levels combined with AMF inoculation (AP50 and AG50). In fungal communities (Figure 5b), *Ascomycota* was the dominant phylum. Its relative abundance was highest in the CK groups and decreased in the AMF powder treatments under higher fertilization (AP100, AP75), accompanied by increased abundances of *Mortierellomycota* and *Basidiomycota*. Comparable compositional changes were observed in the corresponding AMF granular treatments (AG100, AG75). Treatments with lower fertilization levels (AP50, AG50) resulted in less pronounced changes in fungal phylum composition compared to the high-fertilizer AMF treatments.

### 3.6. Differences and Biomarker Analysis of Bacterial and Fungal Communities

**Figure 6:**
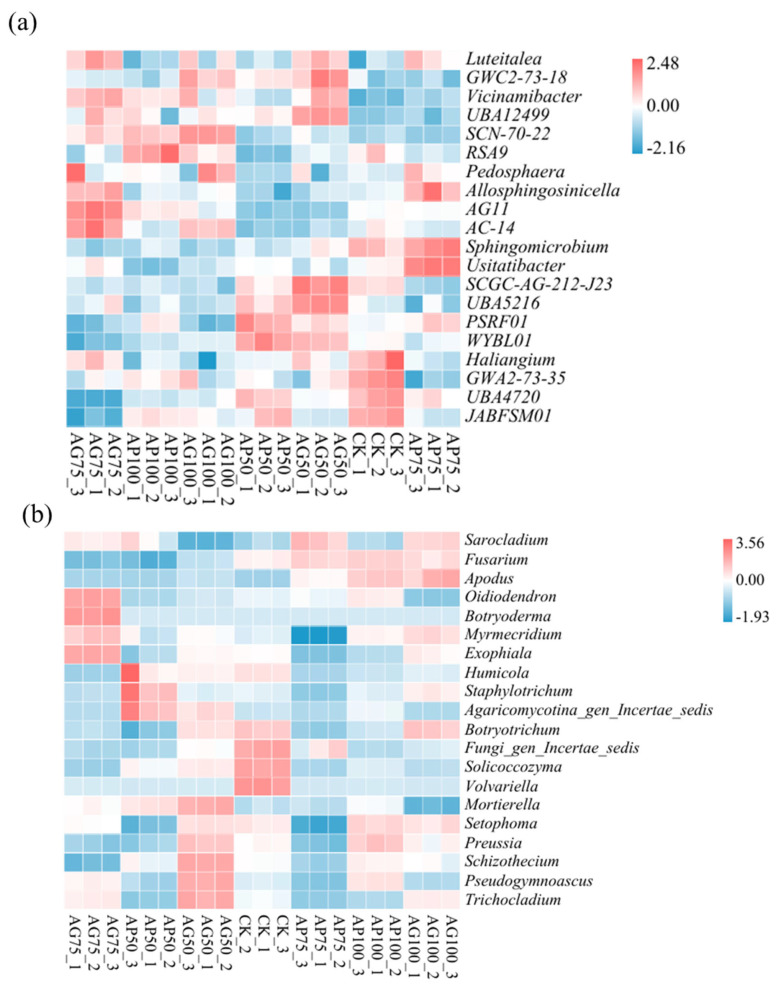
Heatmap analysis of top 20 genera abundance in soil bacterial (**a**) and fungal (**b**) communities under different fertilization treatments.

To compare inter-sample compositional divergence and visualize taxon abundance distribution patterns, heatmap analysis was performed on the top 20 genera by mean abundance. Bacterial characteristic microbial thermograms are shown in Figure 6a. The control (CK) was dominated by *Haliangium*, *GWA2-73-35*, *UBA4720*, and *JABFSMO1* (oligotrophic taxa). AP100 was enriched with *Vicinamibacter* (P-solubilizing), *UBA12499*, *SCN-70-22*, and *RSA9*. AP75 was characterized by *Allosphingosinicella* (aromatic degrader), *Sphingomicrobium*, and *Usitatibacter*. AG75 featured *Pedosphaera*, *Allosphingosinicella*, *AGIi*, and *AC-14* (soil structure modifiers).

A thermogram of microorganisms characterized by observational fungi is shown in Figure 6b. The control (CK) showed elevated abundances of *Botryotrichum*, *Solicoccozyma*, *Fusarium*, and *Volvariella*. AP75 was co-dominated by *Sarocladium* and *Fusarium*. AG50 was enriched in *Mortierella* (P-solubilizing), *Setophoma*, *Preussia*, *Schizothecium*, *Pseudogymnoascus*, and *Trichocladium*.

### 3.7. Correlations Between Bacterial and Fungal Communities and Soil Properties

**Figure 7:**
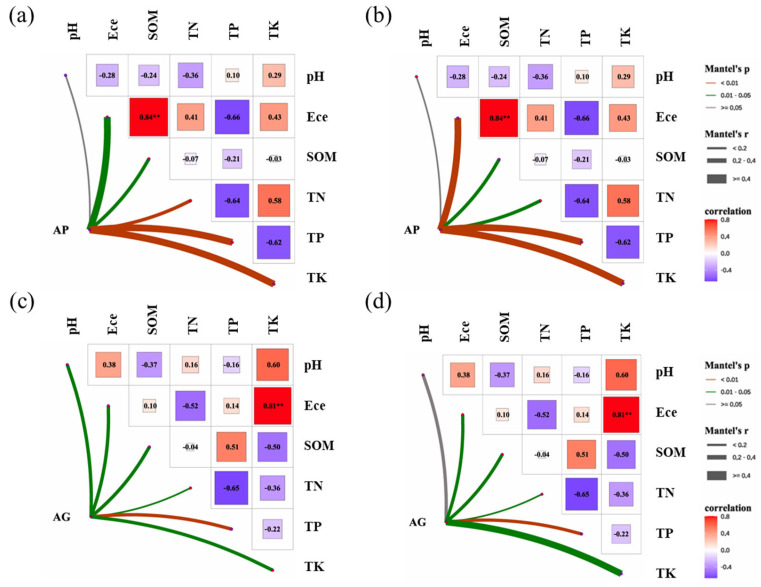
Mantel correlations of bacterial (**a**,**c**) and fungal (**b**,**d**) communities with soil properties. Line features denote Mantel r and significance. Asterisks indicate significance levels: ** *p* < 0.01.

The Mantel test was employed to assess correlations between soil microbial community composition and environmental variables (Figure 7). For bacterial communities, composition exhibited significant correlations with several soil properties, with varying correlation strengths observed across different inoculant types. Within the AMF powder treatment series AP, bacterial community composition exhibited strong positive correlations with total nitrogen (TN: r = 0.65, *p* = 0.003), total phosphorus (TP: r = 0.72, *p* = 0.001), and total potassium (TK: r = 0.61, *p* = 0.007). Weaker yet significant associations were observed with electrical conductivity (ECe: r = 0.48, *p* = 0.022) and soil organic matter (SOM: r = 0.45, *p* = 0.033). In contrast, within the AMF powder-treated series (AG), bacterial community composition showed strong correlations only with total phosphorus (TP) (*p* < 0.01), exhibiting no significant associations with other measured soil properties (*p* > 0.05). Fungal communities exhibited distinct patterns: In the AMF powder-treated group (AP), fungal community composition showed strong correlations with electrical conductivity (ECe), total phosphorus (TP), and total potassium (TK) (all *p* < 0.01), and significant correlations with soil organic matter (SOM) and total nitrogen (TN) (*p* < 0.05). Similarly, in the AMF granular series (AG), fungal community composition showed a strong correlation with total phosphorus (TP) (*p* < 0.01) and a significant correlation with ECe, SOM, TN, and TK (*p* < 0.05).

### 3.8. Economic Benefit Analysis

**Figure 8:**
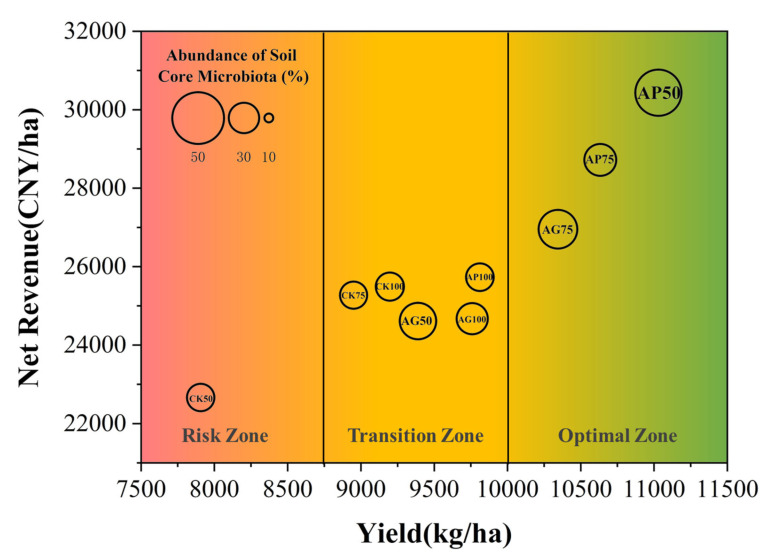
Economic benefit analysis of different treatments. Bubble size represents the relative proportion of core beneficial microbiota; color indicates the agronomic recommendation level. Bubble labels correspond to treatment codes (Table 2).

Based on Chinese market research (2024), the unit prices were 3.5 CNY/kg for chemical fertilizer, 7111 CNY/kg for AMF powder, and 167 CNY/kg for the granular formulation, with application rates of 0.225 kg/ha and 15 kg/ha, respectively. The purchase price of maize was 3 CNY/kg. Detailed cost data are provided in Appendix A, Table A2. Economic benefit analysis revealed that under reduced fertilization conditions, AMF powder formulations yielded significantly higher benefits than granular formulations (Figure 8). In the figure, “beneficial microbial groups” refers to the sum of the relative abundances of specific taxa identified as functionally beneficial in this study, including the phosphate-solubilizing bacterial genus *Vicinamibacter*, the phosphate-solubilizing fungal genus *Mortierella*, and the entire phylum Actinobacteria due to its broad role in organic matter decomposition and nutrient cycling.

Specifically, the combination of 50% chemical fertilizer with the powder inoculant (AP50) achieved the highest net profit (30,435 CNY/ha). This represents a 34.2% increase in profit and a 39.5% increase in yield compared to the non-inoculated control at the same fertilizer reduction level (CK50). Furthermore, it achieved a benefit–cost ratio of 11.5 with the lowest total production cost (2650 CNY/ha). The powder formulation consistently enhanced both yield and profit under 50–75% fertilizer reduction, with the AP75 treatment yielding 10,632 kg/ha. In contrast, the granular formulation, due to its higher cost (a 56.3% increase) and limited yield improvement, resulted in a net profit for the full-rate fertilizer treatment (AG100) that was even lower than that of the non-inoculated control. Solely reducing chemical fertilizer by 50% (CK50) led to the lowest profit (22,669 CNY/ha).

**Table 2:** List of standard codes for different agronomic fertilizer applications.

Agronomic Approach	AMF Powder	AMF Granular	CK
50 percent chemical fertilizer	AP50	AG50	CK50
75 percent chemical fertilizer	AP75	AG75	CK75
100 percent chemical fertilizer	AP100	AG100	CK100

## 4. Discussion

Our field experiments demonstrate that under low-fertility conditions, arbuscular mycorrhizal fungal inoculation significantly alters nutrient dynamics within the plant–soil system (Figure 1). Compared to the fully fertilized control (CK100), soils inoculated with AMF exhibited reduced total nitrogen content and increased total phosphorus content. This pattern aligns with established mechanisms: AMF hyphae compete with roots for nitrogen uptake [22,23], while enhancing phosphorus availability through acid phosphatase secretion and hyphal exploration [24,25]. The increase in total phosphorus was particularly pronounced in the moderate fertilizer reduction combined with AMF powder treatment groups (AP50, AP75). Crucially, these changes were accompanied by stable or enhanced maize yields (Figure 2), indicating that AMF inoculation not only altered soil nutrient pools but also effectively enhanced plant nutrient acquisition capacity, thereby compensating for reduced fertilizer inputs [9]. The positive correlation between yield and both soil organic matter (SOM) and total phosphorus (Figure 2c) further corroborates the role of AMF in coupling organic matter dynamics with phosphorus cycling, a mechanism potentially involving actinomycin-associated soil proteins [26].

The efficacy of AMF is initiated by root colonization, which varied distinctly between formulations and fertilizer levels (Figure 2a). Powder formulation colonization rates were highest under 50% fertilizer reduction (AP50: 85.3%) and declined with increasing fertilizer, consistent with the “carbon-for-nutrient” trade model where moderate phosphorus stress promotes symbiotic investment [27]. In contrast, granular formulation colonization exhibited a hump-shaped response, peaking at 75% reduction (AG75). We hypothesize this pattern arises from an interaction between nutrient stress and formulation properties [9]. Under severe reduction (AG50), plant demand is high, but the slower spore germination and hyphal growth of granules may delay establishment during critical early growth. Under full fertilizer (AG100), low plant dependency suppresses colonization. The AG75 treatment may thus represent a balance where sufficient stress drives symbiosis, and the slow-release properties of granules support persistent colonization activity throughout the season. This highlights formulation release kinetics as a critical factor mediating the plant–symbiont relationship under varying nutrient conditions [28].

Sequencing analyses revealed that fungal communities exhibited a greater response to fertilization and inoculation treatments than bacterial communities, as evidenced by significant alterations in α-diversity indices (Figure 3) and pronounced segregation trends in β-diversity principal coordinate analysis plots (Figure 4) [29]. This heightened sensitivity may stem from arbuscular mycorrhizal fungi, which directly influence fungal communities through mycelial networks and secretions [16,30]. At the phylum level, AMF inoculation (particularly under low-fertilizer treatments) consistently elevated the relative abundance of *Actinobacteria* and *Mollicutes*, while reducing the dominance of *Proteobacteria* and *Ascomycota* observed in uninoculated controls (CK series) (Figure 5) [31]. These alterations indicate that AMF fungi reshaped soil microbial community composition. Genus-level analysis provided insights with greater functional interpretative value (Figure 6). Treatments with high colonization success (e.g., AP50) exhibited enrichment of genera with known beneficial properties, such as the phosphorus-solubilizing bacterium *Vicinamibacter* [32] and the phosphorus-solubilizing fungus *Mortierella* [33,34]. Conversely, the pathogenic fungus Fusarium was more abundant in uninoculated low-fertilizer controls (CK50, CK75). The AG75 treatment group enriched genera such as *Pedosphaera*, frequently associated with complex organic matter decomposition. Mantel analysis indicated total phosphorus as the strongest environmental factor correlating with microbial community composition (Figure 7), directly revealing the link between AMF-mediated enhanced phosphorus availability (Figure 1) and microbial restructuring [35].

We have synthesized our research findings into two distinct pathways centered on formulation, linking management practices to agricultural economic benefits (Table 3): The first is the rapid and efficient pathway (represented by AP50): This powder formulation enables root colonization, establishes mycelial networks, enhances phosphorus uptake efficiency, and recruits readily available phosphorus-solubilizing microorganisms *Vicinamibacter* and *Mortierella* under conditions of 50% fertilizer reduction [36]. This pathway maximizes phosphorus supply, delivering immediate yield increases (14.67%) and net profits (¥30,435/ha). It addresses acute nutrient deficiencies in medium- to low-fertility soils, optimizing annual income. The second is the gradual stabilization pathway (represented by AG75): The granular formulation promotes slow yet sustained colonization at 75% fertilizer reduction [28]. This long-term symbiotic interaction appears to favor microbial communities with enhanced organic matter processing capabilities *Actinobacteria* and *Basidiomycota*. This pathway prioritizes soil organic matter accumulation and ensures sustained nutrient supply, delivering moderate yet stable yield gains (7.18%) while laying foundations for improved soil health [9]. This makes it suitable for systems prioritizing long-term soil quality and resilience, such as continuous cropping systems or high-organic-matter soils.

## 5. Conclusions

This study elucidates the differential regulatory mechanisms and decision pathways of arbuscular mycorrhizal fungal (AMF) formulations in reduced-fertilization systems, offering a microbiome-driven precision strategy for agricultural green transition. The powder formulation (AP50) achieved a 14.67% yield increase and a net profit of 30,435 CNY/ha under 50% fertilizer reduction through rapid root colonization and recruitment of functional microbes. In contrast, the granular formulation (AG75) utilized its slow-release properties to enhance soil organic matter, ensuring system stability and sustained yield gain under 75% reduction. We demonstrate that fungal communities are particularly sensitive to fertilization strategies, and that AMF inoculation can effectively suppress pathogens while enriching functional taxa, thereby overcoming the microbial functional thresholds imposed by fertilizer reduction alone. These findings not only advance the theoretical understanding of bio-inoculant and soil microbiome interactions, but also provide agricultural practitioners, farmers, and policymakers with a dual-mode framework—the “quick-profit” (AP50) and “soil-health” (AG75) options—enabling context-specific implementation for sustainable intensification across diverse soil fertility conditions.

## Figures and Tables

**Table 1:** Initial physicochemical properties of the experimental site soil.

Category	Parameter	Value/Range	Unit
Chemical Properties	pH	8.34 ± 0.12	
	Electrical Conductivity (EC)	0.25 ± 0.03	dS/m
	Soil Organic Matter (SOM)	15.76 ± 2.46	g/kg
	Total Nitrogen (TN)	1.87 ± 0.24	g/kg
	Total Phosphorus (TP)	0.94 ± 0.15	g/kg
	Total Potassium (TK)	46.43 ± 0.55	g/kg
	Alkali-hydrolyzable Nitrogen (AN)	187.4 ± 25.6	mg/kg
	Available Phosphorus (AP)	9.43 ± 1.47	mg/kg
	Available Potassium (AK)	145.2 ± 18.3	mg/kg
	Exchangeable Al	0.12 ± 0.05	cmol^+^/kg
	Available Fe	6.85 ± 1.22	mg/kg
	Available Mn	10.34 ± 2.15	mg/kg
	Available Cu	1.56 ± 0.33	mg/kg
	Available Zn	1.02 ± 0.28	mg/kg
Physical Properties	Bulk Density	1.32 ± 0.08	g/cm^3^
	Total Porosity	50.2 ± 3.5	%
	Field Capacity	24.5 ± 2.1	%
	Soil Texture	Sandy Loam	
	Sand (0.05–2 mm)	62.5	%
	Silt (0.002–0.05 mm)	25.8	%
	Clay (<0.002 mm)	11.7	%

**Table 3:** Recommended classification of agronomic measures under different cropping objectives.

Objectives	Preferred Mode	Economic Indicator	Conditions of Application
Increase in income for the year	AP50	Net income of 30,435 CNY/ha	Medium- to low-fertility soils
soil improvement	AG75	0.8 g/kg annual increase in organic matter	Continuous cropping systems/high organic matter soils
risk avoidance	CK100	Stable returns but high environmental costs	Traditional Conservative Cultivation

## Data Availability

The original contributions presented in this study are included in the article. Further inquiries can be directed to the corresponding author.

## Abbreviations

The following abbreviations are used in this manuscript:

AMF	Arbuscular mycorrhizal fungi
AP50	AMF powder combined with 50% chemical fertilizer treatment
AG50	AMF granular combined with 50% chemical fertilizer treatment
CK50	Treatment with 50% chemical fertilizer without AMF addition
AP75	AMF powder combined with 75% chemical fertilizer treatment
AG75	AMF granular combined with 75% chemical fertilizer treatment
CK75	Treatment with 75% chemical fertilizer without AMF addition
AP100	AMF powder combined with 100% chemical fertilizer treatment
AG100	AMF granular combined with 100% chemical fertilizer treatment
CK100	Treatment with 100% chemical fertilizer without AMF addition
EC	Electrical conductivity
SOM	Soil organic matter
TN	Total nitrogen
TP	Total phosphorus
TK	Total potassium

## Appendix A

**Table A1:** Parameters of soil indicators for different agronomic measures.

Process Group	pH	ECe	SOM	AN	AP	AK
CK_1	7.05	0.25	35.08168	41.72	9.671726	45.12
CK_2	7.3	0.22	34.3938	41.27	11.49665	58.19
CK_3	7.1	0.32	35.08168	64.57	5.788916	72.27
AP100_1	7.25	0.3	35.08168	47.15	5.612425	74.13
AP100_2	7.15	0.28	35.76955	41.63	12.84857	63.88
AP100_3	7.2	0.35	37.1453	43.92	7.730321	60.75
AP75_1	7.35	0.33	35.08168	43.98	15.49594	70.04
AP75_2	7.12	0.34	37.1453	41.82	12.55559	67.95
AP75_3	7.4	0.27	35.08168	56.08	6.141899	53.34
AP50_1	7.5	0.31	26.13929	44.39	9.848217	82.21
AP50_2	7.45	0.29	39.20893	43.95	17.61384	48.88
AP50_3	7	0.23	33.01805	46.23	12.49559	39.73
AG100_1	7.18	0.21	29.134	43.16	11.84822	54.45
AG100_2	7.15	0.3	31.543	32.28	3.26509	68.02
AG100_3	7.24	0.39	23.146	43.99	15.14296	81.88
AG75_1	7.26	0.29	24.563	38.84	16.79139	65.34
AG75_2	7.42	0.36	31.34	62.51	16.49559	56.12
AG75_3	7.08	0.24	27.754	47.09	12.0826	66.09
AG50_1	7.44	0.28	43.143	35	19.59381	47.5
AG50_2	7.03	0.3	45.143	38.5	27.20979	42.5
AG50_3	7.05	0.23	33.175	35	23.4018	72.5

**Table A2:** Analysis of economic benefits of different agronomic measures.

Group	Chemical Fertilizer Cost (CNY/ha)	Microbial Inoculant Cost (CNY/ha)	Total Production Cost (CNY/ha)	Yield (kg/ha)	Gross Return (CNY/ha)	Net Return (CNY/ha)
AP100	2100	1600	3700	9810	29,431	25,731
AP75	1575	1600	3175	10,632	31,896	28,721
AP50	1050	1600	2650	11,028	33,085	30,435
AG100	2100	2500	4600	9758	29,276	24,676
AG75	1575	2500	4075	10,343	31,029	26,954
AG50	1050	2500	3550	9388	28,165	24,615
CK100	2100	0	2100	9196	27,590	25,490
CK75	1575	0	1575	8949	26,849	25,274
CK50	1050	0	1050	7906	23,719	22,669
